# A suspected case of *Clostridium perfringens* sepsis with intravascular hemolysis after transhepatic arterial chemoembolization: a case report

**DOI:** 10.1186/s13256-019-2023-x

**Published:** 2019-04-27

**Authors:** Haruki Uojima, Mie Onoue, Hisashi Hidaka, Naohisa Wada, Yoshiaki Tanaka, Tomoyoshi Inoue, Kousuke Kubota, Takahide Nakazawa, Akitaka Shibuya, Wasaburo Koizumi

**Affiliations:** 1 Department of Gastroenterology, Shonan Kamakura General Hospital, 1370-1 Okamoto, Kamakura, Kanagawa 247-8533 Japan; 2 Department of Gastroenterology, Internal Medicine, Kitasato University School of Medicine, 1-15-1 Kitasato, Minami-ku, Sagamihara, Kanagawa 252-0375 Japan

**Keywords:** Intravascular hemolysis, *Clostridium perfringens*, Transhepatic arterial chemoembolization

## Abstract

**Introduction:**

Sepsis due to *Clostridium perfringens,* one of several clostridial species, is an important cause of massive intravascular hemolysis in patients with underlying malignancies. Chronic liver diseases, immunosuppression, and presence of malignancies were risk factors for *Clostridium perfringens* sepsis. Therefore, *Clostridium perfringens* sepsis should always be considered in patients presenting with liver damage after chemo-embolic therapy for hepatocellular carcinoma. This case report focuses on findings characteristic of an intravascular hemolysis due to *Clostridium perfringens* after transhepatic arterial chemoembolization.

**Case presentation:**

An 83-year-old Japanese man presented to our hospital because of a third recurrence of hepatocellular carcinoma. He had nonalcoholic steatohepatitis-related cirrhosis, and underwent radiofrequency ablation and transhepatic arterial chemoembolization therapy for hepatocellular carcinoma of S4/S8 and S2. He had a medical history of pancreatic carcinoma and underwent pylorus-preserving pancreaticoduodenectomy approximately 5 years ago. Because follow-up computed tomography showed a recurrence of the hepatocellular carcinoma, he underwent transhepatic arterial chemoembolization with a hepatic arterial infusion of 20 mg epirubicin, followed by 4 mL Lipiodol (ethiodized oil). On the sixth day after the procedure, he complained of fever and hematuria with jaundice. Laboratory findings indicated hemolysis and increased inflammatory response. Although we initiated antibiotic therapy combined with surgical debridement for infection after transhepatic arterial chemoembolization, he died within 6 hours. The autopsy showed a 4-cm local necrotic hepatic tumor. The cut surface revealed a tumor with an internal spongiform appearance, which was a pseudocystic and partially necrotic lesion. In addition, a diffuse spread of Gram-positive rods in multiple organs including the heart was histologically confirmed. The culture obtained by fluid aspiration from the hepatic abscess revealed *Clostridium perfringens*. Although the role of *Clostridium perfringens* was never established during the life of this patient, based on the clinical course and the culture from the hepatic abscess at postmortem, intravascular hemolysis secondary to *Clostridium perfringens* sepsis was suspected.

**Conclusion:**

Intravascular hemolysis secondary to *Clostridium perfringens* should always be considered in patients presenting with liver damage after chemo-embolic therapy for hepatocellular carcinoma. Biliary reconstruction is an especially important risk factor for infection.

## Introduction

Sepsis due to *Clostridium perfringens,* one of several clostridial species, is an important cause of massive intravascular hemolysis in patients with underlying malignancies [1, 2]. Although *C. perfringens* sepsis is rare, massive intravascular hemolysis due to *C. perfringens* sepsis can have a particularly rapid fatal clinical course [3]. A literature review showed that chronic liver diseases, immunosuppression, and presence of malignancies were risk factors for *C. perfringens* sepsis [4]. In particular, *C. perfringens* sepsis should always be considered in patients presenting with liver damage after chemo-embolic therapy for hepatocellular carcinoma (HCC). This case report focuses on findings characteristic of an intravascular hemolysis secondary to *C. perfringens* sepsis after transhepatic arterial chemoembolization (TACE).

## Case presentation

**Fig. 1:**
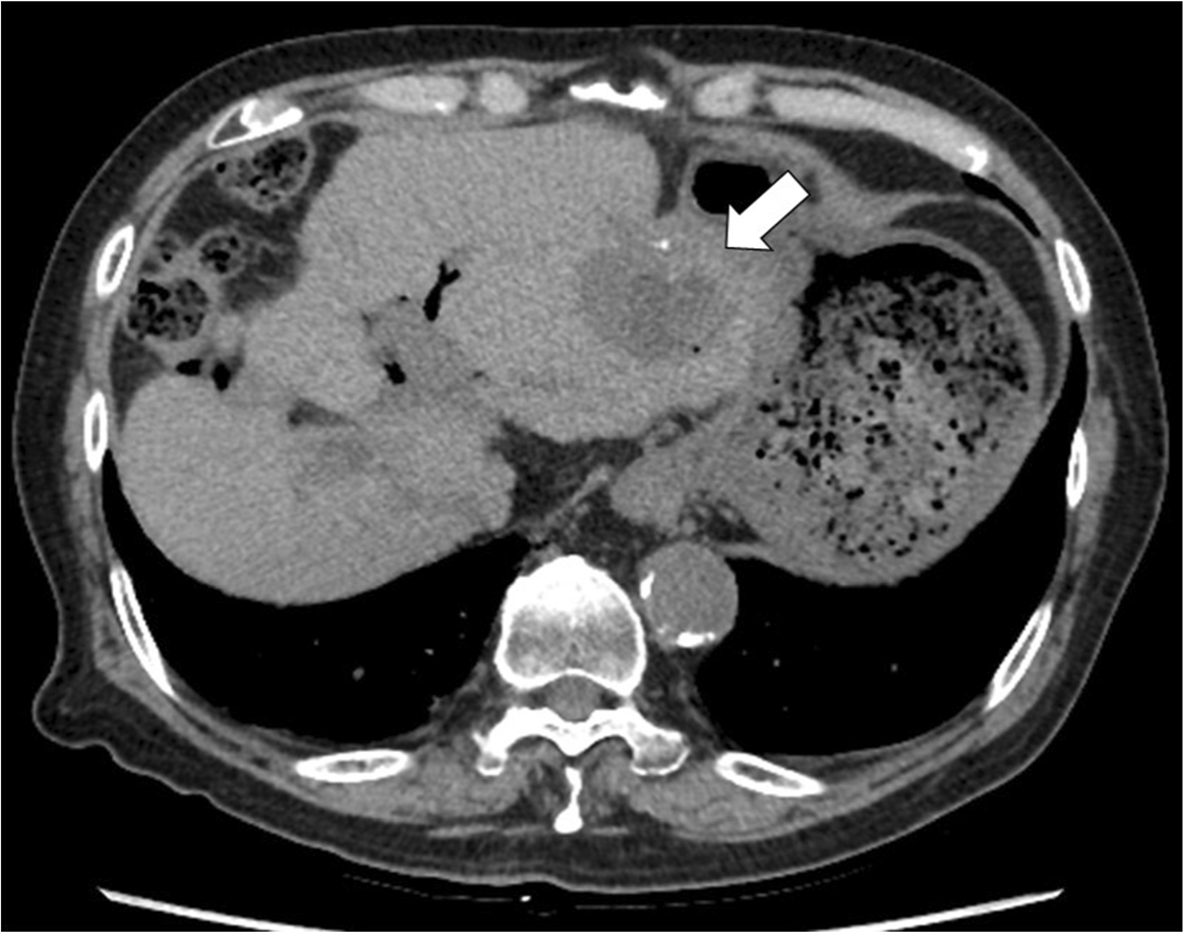
Computed tomography images in the axial plane. A low-density nodule (*white arrow*) in the left lobe is shown. Dynamic computed tomography was difficult to perform in this patient because of decreased renal function

An 83-year-old Japanese man presented to our hospital because of a third recurrence of HCC. He had nonalcoholic steatohepatitis-related cirrhosis, and underwent radiofrequency ablation for a partial HCC of S4/S8 in his liver 3 years ago. Because abdominal computed tomography (CT) revealed multiple HCC of S4/S8 and S2 in his liver 1 year ago, he underwent TACE therapy with an emulsified mixture of Lipiodol (ethiodized oil) and Farmorubicin (epirubicin) together with gelatin sponge particles for multiple tumors. After the second TACE, abdominal CT revealed sufficient Lipiodol (ethiodized oil) retention and the inefficacy of this treatment. However, follow-up CT showed a HCC recurrence in the left lobe 2 months ago. His medical history included reflux esophagitis, hypertension, and pancreatic carcinoma and he underwent pylorus-preserving pancreaticoduodenectomy approximately 5 years ago. His medications included amlodipine 5 mg, candesartan 4 mg, and esomeprazole 20 mg, all once daily. He was nondiabetic, did not smoke tobacco or drink alcohol, and had no history of any drug or food allergies. His family and social history were unremarkable. He appeared well on presentation. His body mass index was 26.2 kg/m^2^, with no noticeable body weight changes. He had an axillary temperature of 36.0 °C, a heart rate of 70 beats/minute, and blood pressure of 118/52 mmHg, with an oxygen saturation of 98% on room air at admission. No conjunctival pallor, icterus, cyanosis, or spider nevi were detectable on physical examination. Cardiovascular and respiratory examinations indicated normal jugular venous pressure and heart sounds, with no detectable murmurs, and normal breath sounds, with no crackle or wheeze. There were no particular abnormal physical findings. Laboratory studies indicated elevated creatinine and α-fetoprotein levels (Table 1). Abdominal ultrasonography showed several hypoechoic masses in his liver; an abdominal plane CT showed multiple lesions with the greatest extent more than 40 mm in the left lobe of his liver (Fig. 1).

**Table 1:** Summary of the laboratory data

		Normal range	Before procedure	The sixth day after the procedure
Complete blood count				
White blood cells	× 10^2/^ μL	30–97	40	291
Neutrophils	%	36.6–79.9	70.1	92
Hemoglobin	g/dL	13.1–17.6	9.3	5.1
Platelet counts	× 10^4/^ μL	12.4–30.5	11.6	11.4
Biochemistry				
Total bilirubin	mg/dL	0.1–1.2	0.7	14
Aspartate aminotransferase	IU/L	12–35	33	1300
Alanine aminotransferase	IU/L	6–40	76	362
Lactate dehydrogenase	IU/L	119–229	235	4523
γ-glutamyl transpeptidase	IU/L	0–48	82	108
Alkaline phosphatase	IU/L	115–359	427	751
Blood-urea-nitrogen	mg/dL	7.4–19.5	32.8	45.9
Creatinine	mg/dL	0.5–1.2	1.35	1.50
Total protein	g/dL	6.4–8.3	6.7	6.0
Albumin	g/dL	3.8–5.2	3.2	2.4
Sodium	mEq/L	135–147	140	132
Potassium	mEq/L	3.4–4.8	4.1	4.7
Ammonia	μg/dL	12–66	102	145
HbA1c	%	4.6–6.2	5.5	
Coagulation				
PT-INR		0.89–1.12	1.04	1.58
APTT	seconds	23.6–31.3	22.9	44.1
Tumor marker				
Alpha-fetoprotein	ng/mL	0–10	36,690	
PIVKA-II	mAU/mL	0–39	3743	
Serology				
Hepatitis B surface antigen			negative	
Hepatitis C virus antibody			negative	

*APTT* activated partial thromboplastin time, *HbA1c* glycated hemoglobin, *PIVKA-II* protein induced by vitamin K absence-II, *PT-INR* prothrombin time-international normalized ratio

**Fig. 2:**
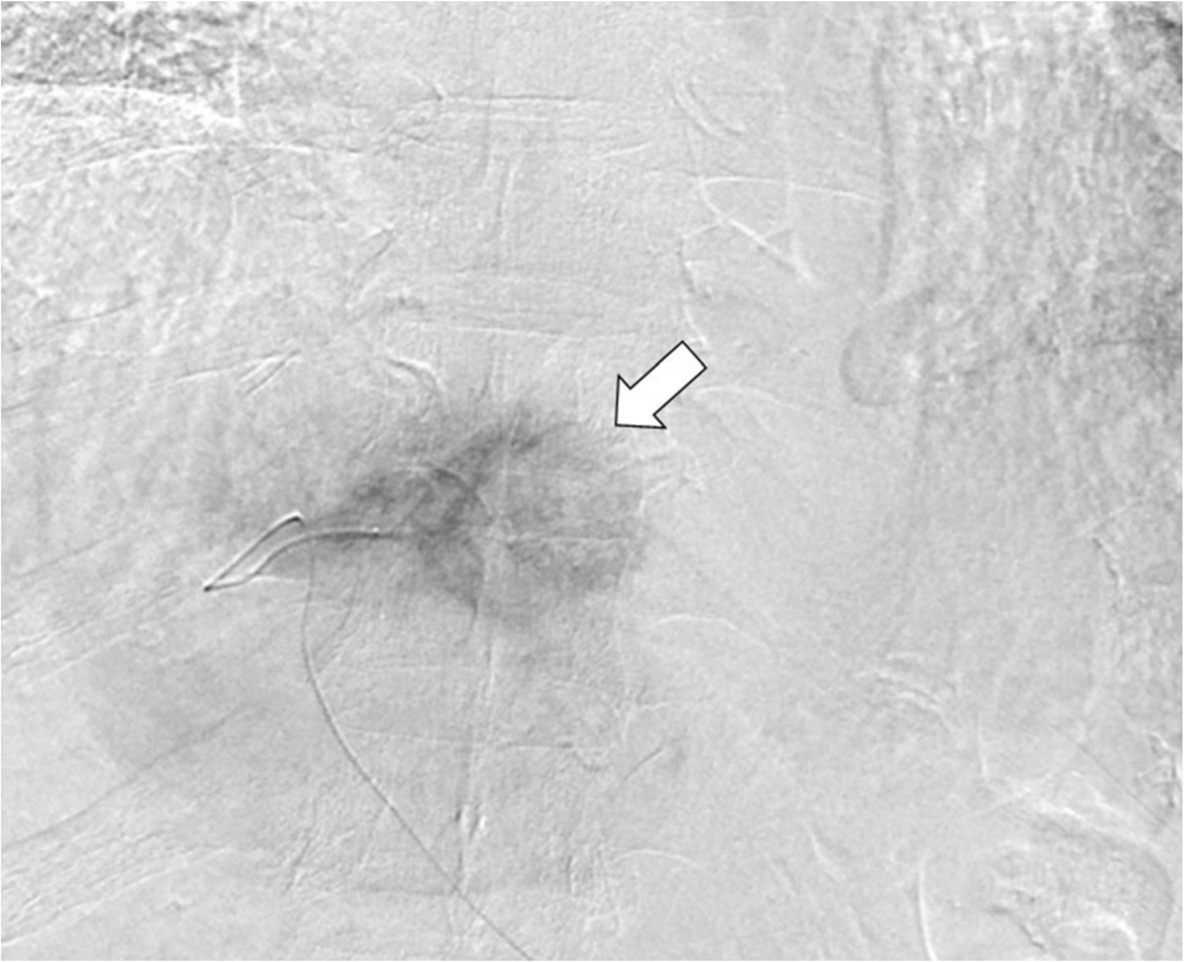
Hepatic angiogram showing a large 40-mm hepatic tumor corresponding to the lesion on plane computed tomography. The hypervascular tumor in the left lobe is depicted as a round mass of contrast opacification (*straight white arrow*) and as being supplied by the left hepatic artery

Owing to our patient’s high risk of liver abscess after TACE because of his medical history of pancreaticoduodenectomy, the treatment course was carefully decided after consultation with our patient and his family. He underwent TACE with a hepatic arterial infusion of 20 mg epirubicin, followed by 4 mL Lipiodol (ethiodized oil) (Fig. 2). A few days after undergoing the procedure, he was generally well except for mild symptoms attributed to postembolization syndrome. Despite antibiotic therapy (cefmetazole 3 grams daily) to prevent infection, he complained of fever, nausea, and hematuria on the sixth day after the procedure. He appeared unwell, severely jaundiced, and extremely restless. When his condition deteriorated, he had an axillary temperature of 39.0 °C, a heart rate of 110 beats/minute, and blood pressure of 90/40 mmHg. He presented with deterioration in hemoglobin levels and renal function, anemia, and a coagulation dysfunction. Furthermore, total bilirubin and direct bilirubin levels increased. Because elevated bilirubin and lactate dehydrogenase due to destruction of red blood cells showed hemolytic anemia, we performed a Coombs test for autoimmune hemolytic anemia to detect the presence of antibodies against red blood cells. However, the results for both the direct and indirect Coombs tests were negative. Based on our patient’s severe clinical course and laboratory data suggestive of hemolysis, intravascular hemolysis secondary to *C. perfringens* sepsis was suspected.

**Fig. 3:**
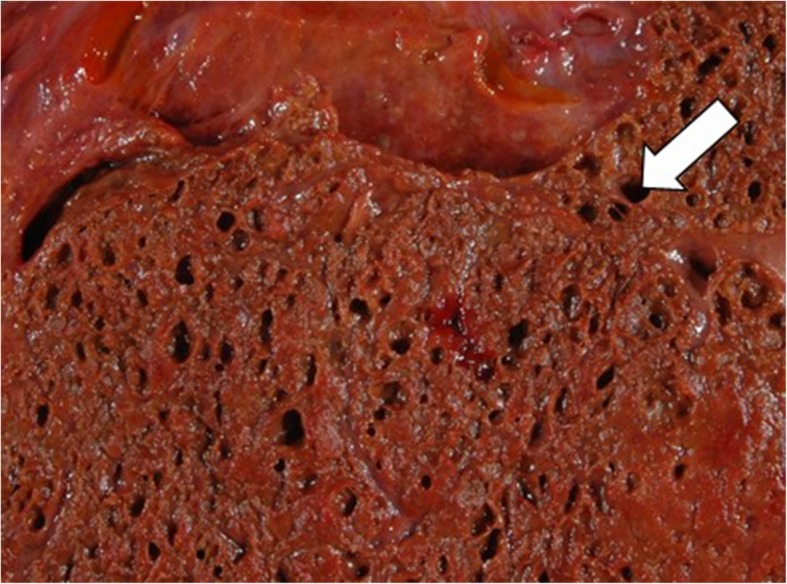
Gross appearance of the liver at autopsy. The cut surface revealed a tumor with an internal spongiform appearance, that of a pseudocystic and partially necrotic lesion measuring 50 mm in the maximum dimension (*white arrowhead*)

**Fig. 4:**
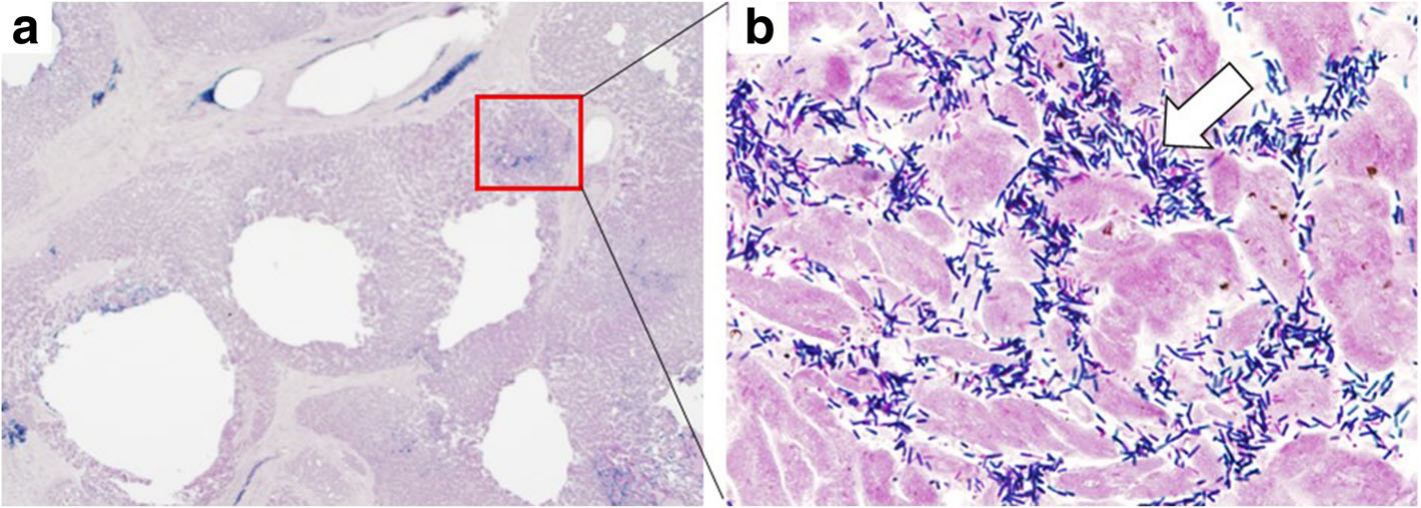
**a** Pathogenesis of multiple small abscesses. These abscesses contained a thin rim of epithelioid histiocytes and other inflammatory cells (Gram stain × 10). **b** Colonies of Gram-positive rods observed on Gram staining (*arrows*) in the heart (Gram stain × 550)

Because the embolic and necrotic lesion after TACE was suspected to be the focus of infection, we initiated antibiotic therapy (piperacillin/tazobactam 4.5 grams and clindamycin 600 mg) combined with surgical debridement. However, he died within 6 hours following unsuccessful cardiopulmonary resuscitation. An autopsy showed a 4-cm local, necrotic, hepatic tumor. The cut surface revealed a tumor with an internal spongiform appearance, which was a pseudocystic and partially necrotic lesion (Fig. 3). In addition, a diffuse spread of Gram-positive rods in multiple organs including the heart was histologically confirmed (Fig. 4). The culture obtained by fluid aspiration from the hepatic abscess revealed *C. perfringens*.

## Discussion

We reported a suspected case of massive intravascular hemolysis due to *C. perfringens* after TACE. In the present case, the role of *C. perfringens* was never established during the life of the patient. However, based on the severe clinical course and the culture obtained by fluid aspiration from the hepatic abscess at postmortem, intravascular hemolysis secondary to *C. perfringens* sepsis was suspected.

*C. perfringens* is an anaerobic Gram-positive rod that is naturally found in the intestines of humans and wild animals [4]. Although *C. perfringens* sepsis is rare, its association with massive hemolysis is well known, with death occurring within hours of presentation in patients similar to the present case [5–7]. The alpha toxin produced by *C. perfringens* is primarily responsible for the disease pathogenesis*.* This toxin*,* which possesses phospholipase C and sphingomyelinase activities, can damage the structural integrity of the red blood cell membrane, leading to spherocytosis and subsequent hemolysis [8, 9].

A review of studies on hemolysis associated with *C. perfringens* in the literature showed that infection was attributed to immunosuppression related to ageing, chronic disease, and presence of malignancies [10]. As the infected focus was commonly the liver and gastrointestinal tract, suspecting *C. perfringens* sepsis after TACE for HCC in liver cirrhosis was reasonable. Therefore, *C. perfringens* sepsis should always be considered in the differential diagnosis of HCC in patients presenting with fever and hemolysis after the procedure.

According to a search of reports in PubMed using the medical subject terms “TACE/transarterial embolization (TAE),” “clostridial sepsis,” and/or “hemolysis,” there were five cases of intravascular hemolysis secondary to *C. perfringens* sepsis after TACE/TAE [11–13] (Table 2). The median age of patients at presentation was 74 years (range, 70–83 years), with all five patients being male. Some patients had important risk factors for infection in the gastrointestinal tract, such as gastrectomy, or invasive endoscopic procedures, resulting in tissue damage. In particular, TACE for HCC after biliary reconstruction including pancreaticoduodenectomy should be avoided as much as possible because severe liver abscess could frequently occur. The median tumor diameter was 99 mm (range, 40–179 mm). In all cases of infection after TACE, epirubicin, doxorubicin, and oxaliplatin were administered, with epirubicin being the most frequently used.

**Table 2:** Patients with intravascular hemolysis secondary to *Clostridium perfringens* sepsis after transhepatic arterial chemoembolization/transarterial embolization

Case	Reference	Year	Age	Sex	Tumor	Diameter	Liver cirrhosis	POD	Risk factor	ALT			Drug	Treatment	Outcome
										Pre TACE	Post TACE	Hemolysis			
1	[11]	1992	83	Male	HCC	115 mm		1		Normal range			Doxorubicin	Antibiotic	Dead
2	[12]	2010	74	Male	HCC			5	Gastrectomy				Epirubicin	Antibiotic Drainage	Survival
3	[12]	2011	70	Male	HCC	83 mm		2	ERBD	316			Epirubicin	Antibiotic Drainage	Dead
4	[13]	2014	71	Male	HCC	179 mm	+	2		145	1652	2312	5-fluorouracil Oxaliplatin	Antibiotic	Survival
5	Our case	2017	83	Male	HCC	40 mm	+	6	PPPD	76	120	362	Epirubicin	Antibiotic	Dead

*ALT* alanine aminotransferase, *ERBD* endoscopic retrograde biliary drainage, *HCC* hepatocellular carcinoma, *POD* postoperative day, *PPPD* pancreaticoduodenectomy, *TACE* transhepatic arterial chemoembolization

The common symptoms of sepsis associated with hemolysis were hematuria, fever, and hypotension, which began within 1 week (range, 1–6 days) after the procedure. Although some reports did not mention laboratory data, the review showed a decrease in the median hemoglobin level from pre-procedure (11.6 mg/dL) to the occurrence of hemolysis (6.6 mg/dL). An increase in the median total bilirubin level from 1.15 mg/dL to 11.9 mg/dL, an increase in the median aspartate aminotransferase (AST) level from 73 to 1626 IU/L, and an increase in the alanine aminotransferase (ALT) level from 110.5 to 1337 IU/L was observed at hemolysis associated with *C. perfringens*. Of note, a few cases showed that liver function enzyme levels at post-procedure were elevated compared with those at pre-procedure. Liver damage after chemo-embolic therapy for HCC may be a high risk factor for *C. perfringens* infection.

Imaging studies, especially ultrasonography and CT, showed multiple hypovascular lesions, but it was difficult to distinguish hepatic abscesses from tumors because there were no diagnostic features of hepatic abscesses after TACE/TAE for HCC. Therefore, diagnosis was bacteriologically confirmed through identification of the presence of *C. perfringens* in the blood culture or the abscess.

With respect to treatment, all the patients received antibiotic therapy, and two patients underwent surgery. The mortality rate was 60.0%, with a median time to death of 9.7 hours (range, 0–96 hours). In all fatal cases, patients already went into shock or died before a diagnosis and decision to operate could be made. In one patient who survived, surgical drainage in combination with appropriate antibiotic therapy may have improved survival. If there was a perceived infected focus such as a hepatic abscess, the recommended treatment included aggressive surgical drainage together with high-dose antibiotic therapy.

## Conclusion

Intravascular hemolysis secondary to *C. perfringens* should always be considered in patients who present with liver damage after chemo-embolic therapy for HCC. Biliary reconstruction is an especially important risk factor for infection.

## Abbreviations

CT: Computed tomography
HCC: Hepatocellular carcinoma
TACE: Transhepatic arterial chemoembolization
TAE: Transarterial embolization
